# The ATA guidelines of thyrotoxicosis

**DOI:** 10.1186/1756-6614-6-S2-A25

**Published:** 2013-04-05

**Authors:** Roman Junik

**Affiliations:** 1 Department of Endocrinology and Diabetology, Nicolaus Copernicus University in Torun, Collegium Medicum in Bydgoszcz, Poland

## 

The authors of guidelines provide 100 recommendations describing many intensively discussed issues. Among them is hepatotoxicity of PTU and limitation of PTU treatment mainly to the first trimester of pregnancy. Methimazole becomes first line ATD for patients with new onset of Graves’ disease. Graves’ orbitopathy (GO) is classified according to its clinical activity and severity. Deterioration of GO after radioactive iodine (RAI) treatment and beneficial effects of steroids to prevent RAI induced exacerbation of GO are described. All patients with subclinical thyrotoxicosis who are older than 65 years and those who are below age 65 and have risk factors or hyperthyroid symptoms should be treated. It is proposed to measure TSH receptor autoantibodies in pregnant women with a previous history of autoimmune thyroid disease. The information about thyroid ultrasonography with the colour-flow Doppler for the rapid differentiation between the two main types of amiodarone-induced thyrotoxicosis, and its help for accurate treatment of these conditions is provided.

